# A Decreased Level of Soluble Klotho Can Predict Cardiovascular Death in No or Mild Abdominal Aortic Calcification Hemodialysis Patients

**DOI:** 10.3389/fmed.2021.672000

**Published:** 2021-05-17

**Authors:** Hong Cai, Xuying Zhu, Jiayue Lu, Minxia Zhu, Shang Liu, Yaping Zhan, Zhaohui Ni, Leyi Gu, Weiming Zhang, Shan Mou

**Affiliations:** Department of Nephrology, School of Medicine, Renji Hospital, Shanghai Jiao Tong University, Shanghai, China

**Keywords:** soluble Klotho, cardiovascular disease, death rate, abdominal aorta calcification, maintenance hemodialysis

## Abstract

**Background:** Soluble Klotho plays an important role in cardiovascular disease and death in chronic kidney disease (CKD). We assessed the relationship between serum soluble Klotho (sKL) level and outcome in MHD patients.

**Methods:** Soluble Klotho was detected by ELISA. Cox regression analysis and Kaplan-Meier analysis showed the relationship between sKL and cardiovascular disease (CVD) mortality in maintenance hemodialysis (MHD) patients.

**Results:** There were 45 cases (35.2%) of all-cause death and 36 cases (28.1%) of CVD mortality. Multivariate linear regression analysis showed that Log[iPTH] (γ = −0.224, *P* = 0.015) was an independent predictor of sKL level. Cox regression showed that lower sKL was associated with higher CVD mortality rate [OR = 0.401, 95% CI (0.183–0.867), *P* = 0.022]. Kaplan-Meier analysis showed that the CVD mortality rate increased significantly in patients with low sKL (*P* = 0.006). Compared with high sKL patients, low sKL patients with no or mild vascular calcification [aortic calcification score (AACs) ≤ 4] had no significant difference in all-cause mortality rate. The CVD mortality rate was significantly lower in high sKL patients (*P* = 0.004) than in those with low sKL. In the severe calcification group (AACs ≥ 5), all-cause and CVD mortality rates were similar between different sKL groups (*P* = 0.706 and 0.488, respectively). The area under the receiver-operating characteristic curve (AUC) of soluble Klotho for predicting the CVD in MHD patients with AACs ≤ 4 was 0.796 (0.647–0.946, *P* = 0.017), sensitivity was 0.921, and specificity was 0.50 for a cutoff value of 307.69 pg/ml.

**Conclusions:** Lower sKL was associated with higher CVD mortality rate. Lower sKL concentration in MHD patients with no or mild calcification can predict CVD mortality.

## Introduction

Cardiovascular disease (CVD) is one of the main causes of mortality in maintenance hemodialysis (MHD) patients. The CVD mortality rate is 10–20 times higher in MHD patients than in the general population (1). Studies have shown that, in addition to traditional risk factors, some non-traditional risk factors, such as micro-inflammatory state, oxidative stress, protein-energy malnutrition, and imbalances of calcium and phosphorus metabolism are contributing factors in vascular calcification (2).

In recent years, the Klotho gene has received much attention as a novel biomarker that may predict the prognosis of MHD patients. The Klotho gene, originally identified as an aging suppressor gene in mice, encodes for 130 kDa of Klotho protein and is widely expressed in the kidney, parathyroid, and brain. Klotho exists in two forms: membrane-bound and secreted. A secreted form of Klotho of 70 kDa is the product of alternative splicing, which releases the extracellular domain of membrane Klotho into blood, where it functions as a circulating substance that exerts multiple systemic biological actions on distant organs. This cleaved extracellular domain of membrane Klotho is referred to as soluble Klotho (sKL) (3). In chronic kidney disease (CKD), sKL play an important role in cardiovascular disease and death (3–5). However, few studies have been done in MHD patients. In this study, we prospectively observed the relationship between sKL level and prognosis in patients with MHD to investigate the role of sKL in predicting the prognosis in MHD patients.

## Methods

### Patients

**Figure 1:**
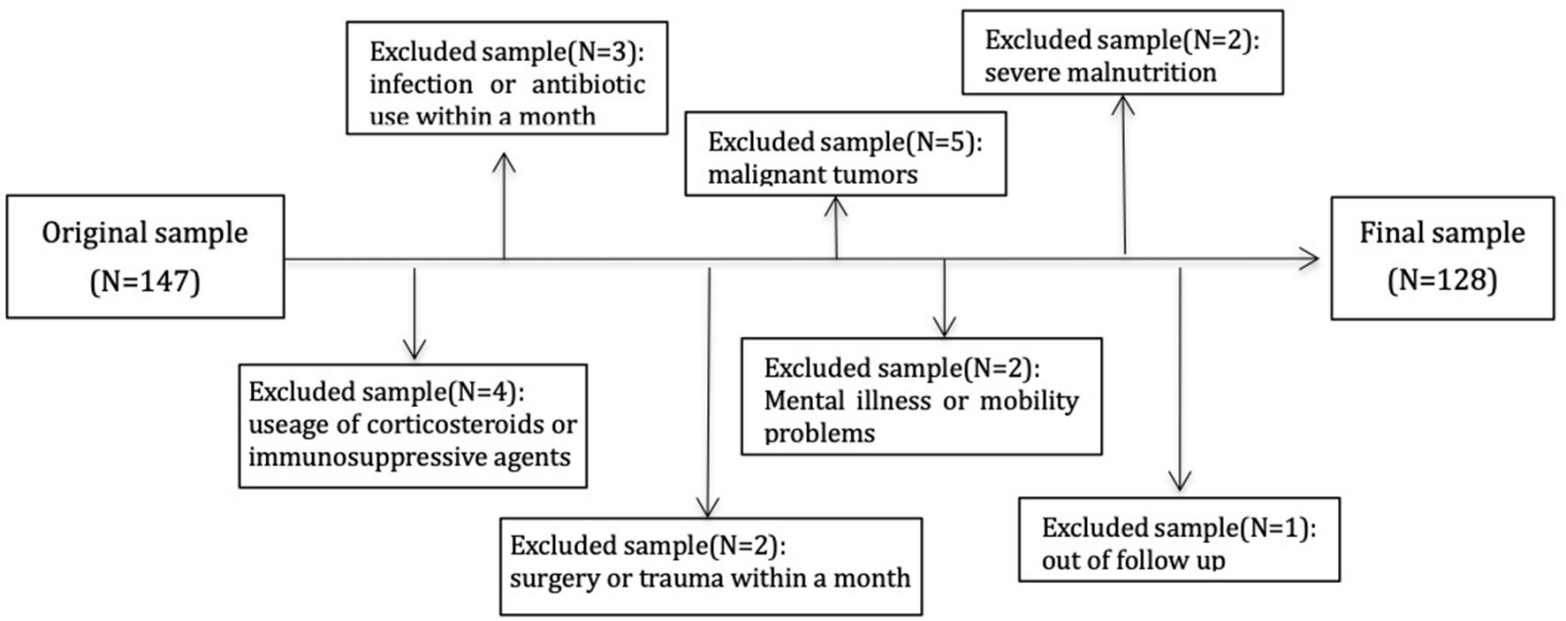
Flowchart describing sample selection. One hundred and forty-seven individual subjects were enrolled and excluded 19 subjects with different reasons. A total of 128 cases were in analysis.

This study protocol was approved by the Ethics Committee on human of Renji Hospital, Jiao Tong University, and all the patients provided written informed consent to participate in this study. The methods were carried out in accordance with the approved guidelines. Patient's inclusion criteria include signing informed consent, older than 18 years, without residual kidney function on MHD between August 2010 and December 2011 with the vintage of longer than 3-month. All the patients were prospectively observed to November 2020. Patients with connective tissue disease, acute infection, trauma, malignant tumor, severe malnutrition, mental illness, and those who needed antibiotics, corticosteroids, immunosuppressive agents, or surgery within a month were excluded. Of 147 patients, 4 were excluded due to usage of corticosteroids or immunosuppressive agents, 3 due to infection or antibiotic use within a month, 2 due to surgery or trauma within a month, 5 due to comorbid malignant tumors, 2 due to severe malnutrition, and 2 due to mental illness or mobility problems, 1 due to out of follow up. A total of 128 individuals were enrolled in the final analytic sample (Figure 1).

Clinical data were recorded, including history of end-stage renal disease cause, concomitant diseases (diabetes mellitus, hypertension, CVD), smoking, medications, pre-dialysis blood pressure, and dialysis duration. In addition, sKL, FGF23, height, weight, and body mass index (BMI) were measured. All patients were dialyzed for 500 ml/min bicarbonate dialysate flow and F80 (Fresenius, Germany) or REXEED (Asahi Kasei Corporation) polysulfide membrane dialyzers. Blood flows were 200–350 ml/min, with dialysis times of 12 h per week. Dialysate calcium 1.5 mmol/l, low dialysate calcium 1.25 mmol/l, dialysate magnesium 0.5 mmol/l. The ultrafiltration target was to achieve the clinically estimated dry weight.

The primary end point was all-cause or CVD death. CVD death was defined as death caused by acute myocardial infarction, pericarditis, cardiac tamponade, cardiomyopathy, coronary atherosclerotic heart disease, arrhythmia, valvular heart disease, pulmonary embolism, cerebral infarction, or cerebral hemorrhage. The cause of death was determined by a physician who did not know the patient's sKL level. When patients died during hospitalization, the cause of death was determined by the attending doctor and recorded in the medical history. If the patients died outside the hospital, the cause of death was based on the death certificate. CVD history was defined as a history of angina pectoris, myocardial infarction, angioplasty, coronary artery disease, peripheral vascular disease, left ventricular hypertrophy, or congestive heart failure (6).

### Plain Radiography of the Abdominal Aorta

A plain lateral radiograph of the abdomen was obtained that included the last two thoracic vertebrae and the first two sacral vertebrae. The aorta was identified as the tubular structure coursing in front of the anterior surface of the spine. A semi-quantitative scoring system was utilized as suggested in the original manuscript by Kauppila et al. (7). Only the segments of abdominal aorta in front of the first to the fourth lumbar vertebrae were considered. Calcific deposits in the posterior and anterior walls of the abdominal aorta adjacent to each lumbar vertebra were assessed separately, using the midpoint of the intervertebral space above and below as the boundaries. Lesions were graded as follows: 0, no aortic calcific deposits; 1, small scattered calcific deposits occupying less than one-third of the length of the corresponding vertebra; 2, moderate quantity of calcific deposits of one-third to less than two-thirds of the corresponding vertebral length; 3, marked calcification of more than two-thirds of the corresponding vertebral length. With this numerical grading, the abdominal aortic calcification score (AACs) could vary from a minimum of 0 to a maximum of 24 points. All radiographs were read and graded by two investigators. The average of the two scores was considered to be the final score. On the basis of the CORD study (8), patients were divided into a none-or-mild calcification group (AACs ≤ 4), or moderate-to-severe calcification group (AACs **≥** 5).

### Laboratory Tests

Biochemical data were obtained using routine laboratory methods. Serum markers relating to mineral metabolism, including total calcium, phosphate, and intact parathyroid hormone (IPTH), were measured, as were hemoglobin, albumin, fasting glucose, C-reactive protein, and lipid levels. Total serum calcium was adjusted for albumin levels using the conversion factor: corrected calcium=calcium+0.02 mmol/L × (40 – albumin).

Blood samples were collected after taking the plain lateral abdominal film and at the time of pre-dialysis. Serum and plasma were separated and frozen at −80°C. Soluble Klotho and FGF23 were measured in plasma using a solid-phase sandwich enzyme- linked immunosorbent assay (ELISA) (Klotho: Immuno-Biological Laboratories, Takasaki, Japan, FGF23: Kainos, Japan).

According to the median distribution of sKL levels, patients were divided into two groups: group I, with sKL levels below the median; group II, sKL levels above the median.

### Statistical Analysis

The Kolmogorov–Smirnov test was used to estimate the Gaussian distribution of the data, and *P* > 0.05 was considered to indicate normal distribution. For normally distributed data, continuous variables were expressed as means and standard deviations, while for skewed data, variables were expressed as medians and interquartile ranges. Categorical variables were expressed as numbers (or percentages). We categorized patients based on the median sKL level within our study population. Differences in demographic data and clinical variables between the groups were analyzed with the independent samples *t*-test or Mann-Whitney *U*-test for continuous variables, and with the chi-square test or Fisher's exact test for categorical variables. Cox regression analysis was performed to assess whether sKL level was a risk factor for CVD death in MHD patients. A Kaplan-Meier survival curve was used to analyze the relationship between the sKL level and MHD, AACs, and CVD death. ROC curves under the curve (AUC) of sensitivity and specificity were used to predicting the risk of CVD and all-cause mortality. *P* < 0.05 was considered statistically significant. SPSS 15.0 (SPSS Inc., Chicago, IL, USA) was used for all statistical analyses and figures.

## Results

### Patient Characteristics

Among the 128 MHD patients, 72 were men, the mean age was 58.29 ± 13.68 years, and the mean dialysis duration was 78.0 (28.0, 121.5) months. There were 45 (35.2%) all-cause deaths, and 36 (28.1%) CVD deaths. Based on the median sKL level, the patients were divided into two groups, with group I having sKL ≤ 567.8 ng/L, and group II having sKL > 567.8 ng/L.

Table 1 shows the basic demographic characteristics. The low sKL group had higher AACs and higher CVD mortality (*P* = 0.045 and 0.001, respectively). The results showed no significant differences in basic demographic data, all-cause mortality, and laboratory data between the groups.

**Table 1:** Baseline characteristics of community-living individuals and laboratory data by median of serum Klotho.

	**All (*n* = 128)**	**Soluble Klotho median**, **Klotho range (pg/mL)**		***P-*value**
		**Low sKL groupsKL ≤ 567.8 (*n* = 64)**	**High sKL group sKL > 567.8 (*n* = 64)**	
Age (years, x ± s)	58.29 ± 13.68	57.13 ± 14.43	59.38 ± 12.86	0.354
Male (*n*, %)	72 (56.3)	41 (64.1)	31 (48.4)	0.108
Smoking (*n*, %)	86 (67.2)	45 (70.3)	41 (64.1)	0.573
Diabetes (*n*, %)	30 (23.4)	17 (26.6)	13 (20.3)	0.532
CVD history, *n* (%)	62 (48.4)	32 (50.0)	30 (46.9)	0.860
Hypertension, *n* (%)	105 (82.0)	52 (81.3)	53 (82.8)	1.000
Primary disease (%)				
CGN	44 (34.4)	25 (39.1)	19 (29.7)	0.352
DKD	10 (7.8)	3 (4.7)	7 (10.9)	0.324
HTN	11 (8.6 )	5 (7.8)	6 (9.4)	1.000
Others	64 (50.0)	37 (57.8)	27 (42.2)	0.111
Dialysis duration [months, *M*(1/4,3/4)]	78.0 (28.0, 121.5)	78.0 (22.0, 122.0)	76.0 (30.0, 117.75)	0.977
Follow up (months)	120.0 (69.0–120.0)	120.0 (64.5–123.0)	120.0 (71.75–120.0)	0.679
BMI [kg/m^2^, x ± s]	21.04 ± 3.03	20.95 ± 3.22	21.14 ± 2.83	0.718
hsCRP [mg/L, *M*(1/4,3/4)]	1.71 (0.83, 4.25)	1.97 (0.80, 5.89)	1.46 (0.87, 3.47)	0.365
Kt/v (x ± s)	1.73 ± 0.36	1.73 ± 0.34	1.72 ± 0.37	0.794
TC (mmol/L, x ± s)	4.36 ± 1.22	4.28 ± 1.05	4.46 ± 1.31	0.388
HDL (mmol/L, x ± s)	1.06 ± 0.44	1.04 ± 0.45	1.07 ± 0.44	0.735
TG [mmol/L, *M*(1/4,3/4)]	1.44 (1.02, 2.36)	1.32 (0.98, 1.94)	1.68 (1.13, 2.70)	0.045
LDL [mmol/L, *M*(1/4,3/4)]	2.26 (1.67, 2.97)	2.16 (1.67, 2.77)	2.39 (1.67, 3.12)	0.160
Hb (g/L, x ± s)	110.35 ± 16.88	108.59 ± 17.10	112.42 ± 16.68	0.203
Hct (%, x ± s)	0.34 ± 0.05	0.33 ± 0.05	0.34 ± 0.05	0.418
Scr (mmol/L, x ± s)	1070.16 ± 252.29	1070.51 ± 257.42	1069.76 ± 248.41	0.987
Adjust Ca (mmol/L, x ± s)	2.38 ± 0.28	2.38 ± 0.28	2.39 ± 0.29	0.823
*P* (mmol/L, x ± s)	2.03 ± 0.58	1.97 ± 0.58	2.08 ± 0.58	0.307
IPTH [ng/L, *M*(1/4,3/4)]	394.0 (169.0, 667.0)	347.0 (154.5, 703.5)	437.5 (187.5, 664.75)	0.462
Alb (g/L, x ± s)	39.43 ± 4.87	39.50 ± 5.03	38.92 ± 4.46	0.494
FGF23 [ng/l, *M*(1/4,3/4)]	6777.97 (2061.98, 9895.29)	5977.77 (1506.80, 9999.71)	7537.41 (2914.11, 9512.51)	0.388
sKlotho [pg/ml, *M*(1/4,3/4)]	567.82 (364.76, 804.19)	387.19 (233.51, 503.06)	819.12 (704.67, 1135.81)	<0.001
AACs [*M*(1/4,3/4)]	4 (0, 11)	8 (0, 13.5)	3 (0, 9)	0.045
SBP (mmHg, x ± s)	139.45 ± 20.68	139.42 ± 20.16	139.52 ± 21.53	0.979
DBP (mmHg, x ± s)	74.67 ± 13.02	75.68 ± 13.39	73.97 ± 12.78	0.460
MBP (mmHg, x ± s)	96.28 ± 13.44	96.93 ± 13.58	95.53 ± 13.35	0.559
Calcium carbonate (*n*, %)	113 (88.3)	60 (93.8)	53 (82.8)	0.097
ACEI/ARB (*n*, %)	79 (61.7)	42 (65.6)	37 (57.8)	0.467
Calcidiol (*n*, %)	56 (43.8)	31 (48.4)	25 (39.1)	0.725
All-cause death (*n*, %)	45 (35.2)	28 (43.8)	17 (26.6)	0.064
CVD death (*n*, %)	36 (28.1)	27 (42.2)	9 (14.1)	0.001

*The low sKL group had higher AACs and higher CVD mortality compared with high sKL group. The results showed no significant differences in basic demographic data, all-cause mortality, and laboratory data between the groups. CGN, Chronic glomerulonephritis; DKD, Diabetes kidney disease; HTN, Hypertensive nephrosclerosis; hsCRP, Hypersensitive C-reactive protein; TG, Triglyceride; TC, Cholesterol; HDL, High-density lipoprotein; LDL, Low density lipoprotein; Hb, Hemoglobin; Hct, Hematocrit; Scr, Serum creatinine; Ca, Calcium; P, Phosphorus; iPTH, Immunoreactive parathyroid hormone; Alb, Albumin; FGF23, Fibroblast growth factor 23; AACs, Abdominal aortic calcification score; SBP, Systolic blood pressure; DBP, Diastolic blood pressure; MBP, Mean blood pressure*.

### Correlation of sKL and Clinical Laboratory Data

Pearson correlation analysis showed that patients' sKL levels were inversely correlated with Log[iPTH] (γ = −0.205, *P* = 0.021), AACs (γ = −0.213, *P* = 0.015), dialysis duration (γ = −0.152, *P* = 0.025), and age (γ = −0.174, *P* = 0.048) (Table 2). Multivariate linear regression analysis showed that Log[iPTH] (γ = −0.224, *P* = 0.015) was an independent predictor of sKL level.

**Table 2:** The relationship between sKL and clinical indicators.

	**sKlotho**	**FGF23**	**Age**	**Dialysis duration**	**MBP**	**Alb**	**Hb**	**LDL**	**Adjust Ca**	***P***	**hsCRP**	**LogiPTH**	**AAC**
sKlotho	1	−0.032	−0.174	−0.152	−0.032	−0.052	0.061	0.001	0.014	0.001	0.079	−0.205	−0.213
	–	0.724	0.048	0.025	0.716	0.561	0.491	0.993	0.876	0.995	0.372	0.021	0.015
FGF23		1	−0.204	0.382	−0.080	0.109	0.075	−0.051	0.508	0.470	−0.055	0.316	0.237
		–	0.025	0.001	0.381	0.235	0.412	0.583	0.001	0.001	0.550	0.001	0.009
Age			1	−0.161	−0.188	−0.355	−0.091	0.149	−0.129	−0.275	0.164	−0.241	0.227
			–	0.068	0.033	0.001	0.303	0.094	0.146	0.002	0.064	0.006	0.010
Dialysis duration				1	−0.162	−0.082	0.181	−0.164	0.248	0.138	−0.009	0.316	0.251
				–	0.066	0.357	0.040	0.064	0.005	0.118	0.923	0.001	0.004
MAP					1	0.135	−0.004	0.043	0.019	−0.014	0.083	0.013	−0.109
					–	0.126	0.969	0.633	0.829	0.879	0.348	0.886	0.219
Alb						1	0.06	−0.149	0.239	0.123	−0.277	0.105	−0.094
						–	0.499	0.094	0.006	0.166	0.001	0.244	0.287
Hb							1	−0.095	0.026	0.070	−0.093	0.037	0.768
							–	0.286	0.767	0.432	0.295	0.678	0.129
LDL								1	−0.134	0.153	0.030	−0.124	0.033
								–	0.131	0.084	0.733	0.169	0.708
Adjust Ca									1	0.036	0.016	−0.032	−0.018
									–	0.684	0.854	0.719	0.839
P										1	−0.208	0.298	0.104
										–	0.018	0.001	0.239
hsCRP											1	−0.016	0.039
											–	0.855	0.658
LogiPTH												1	0.200
												–	0.025
AACs													1
													–

*sKL levels were inversely correlated with log[IPTH], AACs, dialysis duration and age. Multiple liner regression analysis showed that Log[IPTH] was an independent risk factor for sKL level. MBP, Mean blood pressure; Alb, Albumin; Hb, Hemoglobin; LDL, Low density lipoprotein; Ca, Calcium; P, Phosphorus; hsCRP, Hypersensitive C-reactive protein; iPTH, Immunoreactive parathyroid hormone*.

### Relationship Between sKL Level and Prognosis in Patients With MHD

**Figure 2:**
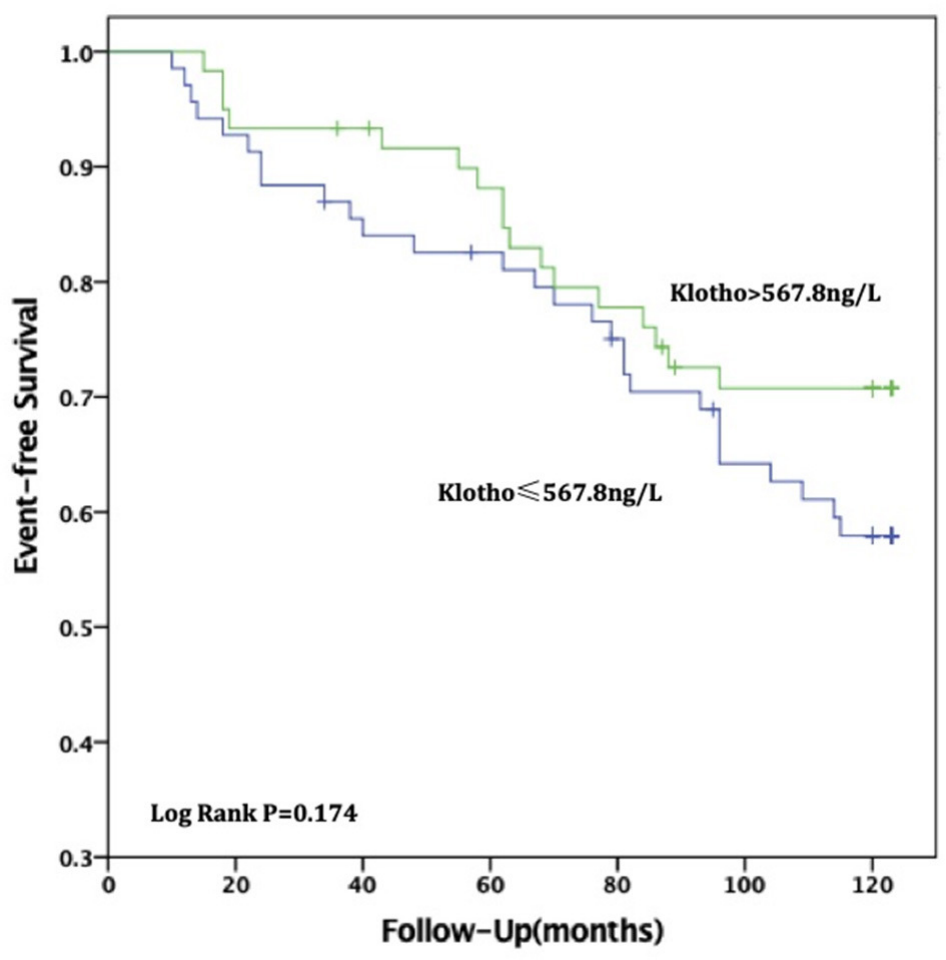
Relationship between soluble Klotho level and all-cause mortality in MHD patients. For all-cause death, patients in high soluble Klotho level (sKl > 567.8 ng/L) had a higher survival time than in the low soluble Klotho level (sKl ≤ 567.8 ng/L). But Kaplan-Meier analysis with log-rank test revealed no significant difference between groups (*P* = 0.174).

**Figure 3:**
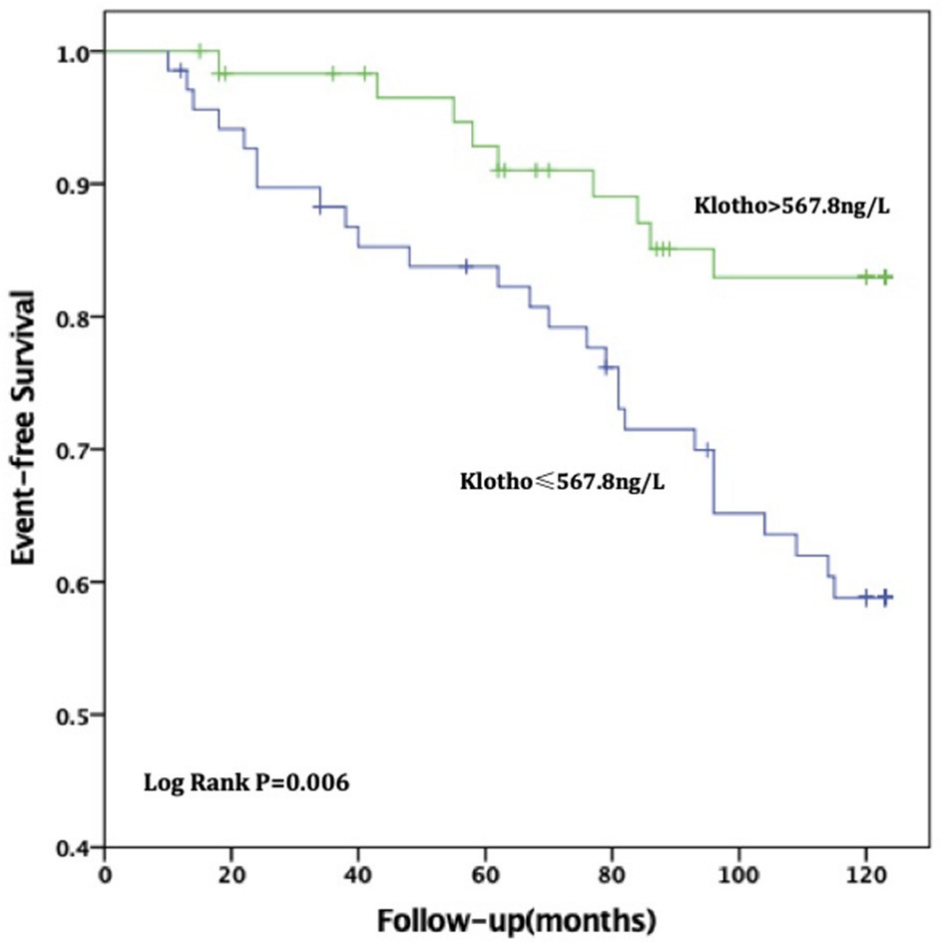
Relationship between soluble Klotho level and CVD mortality in MHD patients. For CVD death, patients in high soluble Klotho level (sKl > 567.8 ng/L) had a significant long survival time than in the low soluble Klotho level (sKl ≤ 567.8 ng/L). Kaplan-Meier analysis with log-rank test revealed a significant difference between groups (*P* = 0.006).

For all-cause deaths, patients with high sKL levels had higher survival times than those in the low sKL group, but the difference was not statistically significant [(97.51 ± 4.54) months vs. (104.06 ± 4.24) months, *P* = 0.174]. For CVD deaths, the group with high sKL levels had significant longer survival times than the low sKL level group [(98.79 ± 4.42) months vs. (113.24 ± 3.17) months, *P* = 0.006] (Figures 2, 3).

### Analysis of Risk Factors for CVD Death in MHD Patients

Univariate analysis showed increased sKL levels to correlate with a reduction in CVD death in MHD patients. Cox regression analysis showed that sKL level (OR = 0.401, 95% CI 0.183–0.867, *P* = 0.022), age (OR = 2.176, 95% CI 1.074–4.406, *P* = 0.031), male (OR = 5.445, 95% CI 1.484–19.972, *P* = 0.011), and levels of hemoglobin (OR = 0.396, 95% CI 0.187–0.840, *P* = 0.016) and AAC score (OR = 3.100, 95% CI 1.421–6.764, *P* = 0.004) to be independent risk factors for CVD death in MHD patients after adjusting for demographics and biochemical indexes (Table 3).

**Table 3:** Analysis of risk factors for CVD death in MHD patients (COX regression analysis).

	**Unadjusted**			**Adjusted1**			**Adjusted2**		
	**OR**	**95% CI**	***P***	**OR**	**95% CI**	***P***	**OR**	**95% CI**	***P***
sKlotho	0.362	0.170–0.769	0.008	0.333	0.156–0.712	0.005	0.401	0.183–0.867	0.022
Age				2.925	1.466–5.836	0.014	2.176	1.074–4.406	0.031
Male				2.852	1.239–6.565	0.014	5.445	1.484–19.972	0.011
Dialysis duration				0.970	0.669–1.406	0.872	1.003	0.596–1.689	0.990
Smoking				1.327	0.627–2.808	0.460	0.809	0.279–2.341	0.695
DM				1.449	0.678–3.099	0.339	1.570	0.553–4.452	0.397
FGF23							1.052	0.356–3.110	0.927
Kt/V							1.796	0.683–4.724	0.235
iPTH							1.164	0.501–2.705	0.725
P							0.996	0.414–2.397	0.993
Adjusted Ca							1.338	0.478–3.748	0.580
Alb							0.855	0.246–2.968	0.805
Hb							0.396	0.187–0.840	0.016
hsCRP							1.497	0.647–3.464	0.346
TG							0.981	0.398–2.421	0.967
TC							0.929	0.263–3.279	0.909
HDL							2.027	0.990–4.1154	0.053
LDL							1.551	0.433–5.550	0.500
AACs							3.100	1.421–6.764	0.004

*Statistical model used COX regression analysis for the risk of CVD death in MHD patients adjusting for demographic data and clinical data. Soluble Klotho level was associated with CVD death. This finding remained consistent in models that adjusted for age and sex, demographic data and clinical data (set sKlotho> 567.8 pg/mL = 1, sKlotho ≤ 567.8 pg/ml = 0; Age ≤ 58.29(y) = 0, Age > 58.29(y) = 1; Male = 1, Female = 0; dialysis duration > 78.0(m) = 1, dialysis duration ≤ 78.0(m) = 0; Smoking = 1, no smoking = 0; Diabetes = 1, non-diabetes = 0; FGF23 > 6777.97 ng/l = 1, FGF23 ≤ 6777.97 ng/l = 0; Kt/v > 1.73 = 1, Kt/v ≤ 1.73 = 0; iPTH > 394.0 ng/l = 1, iPTH ≤ 394.0 ng/l = 0; P > 2.03 mmol/l = 1, P ≤ 2.03 mmol/l = 0; Adjusted Ca > 2.38 mmol/l = 1, Adjusted Ca ≤ 2.38 mmol/l = 0; Alb > 39.43 g/l = 1, Alb ≤ 39.43 g/l = 0; Hb ≤ 110.35 (g/l) = 0, Hb > 110.35(g/l) = 1; hsCRP > 1.71 mg/l = 1, hsCRP ≤ 1.71 mg/l = 0; TG ≤ 1.44 mmol/l = 0, TG > 1.44 mmol/l = 1; TC ≤ 4.36 mmol/l = 0, TC > 4.36 mmol/l = 1; HDL ≤ 1.06 mmol/l = 0, HDL > 1.06 mmol/l = 1; LDL ≤ 2.26 mmol/l = 0, LDL > 2.26 mmol/l = 1; AACs > 4 = 1, AACs ≤ 4 = 0). hsCRP, Hypersensitive C-reactive protein; TG, triglyceride; TC, Cholesterol; HDL, High-density lipoprotein; LDL, Low density lipoprotein; Hb, Hemoglobin; Ca, Calcium; P, Phosphorus; iPTH, Immunoreactive parathyroid hormone; Alb, Albumin; FGF23, Fibroblast growth factor 23; AACs, Abdominal aortic calcification score*.

### The Relationship Between AACs, sKL Level, All-Cause Death and CVD Death

**Figure 4:**
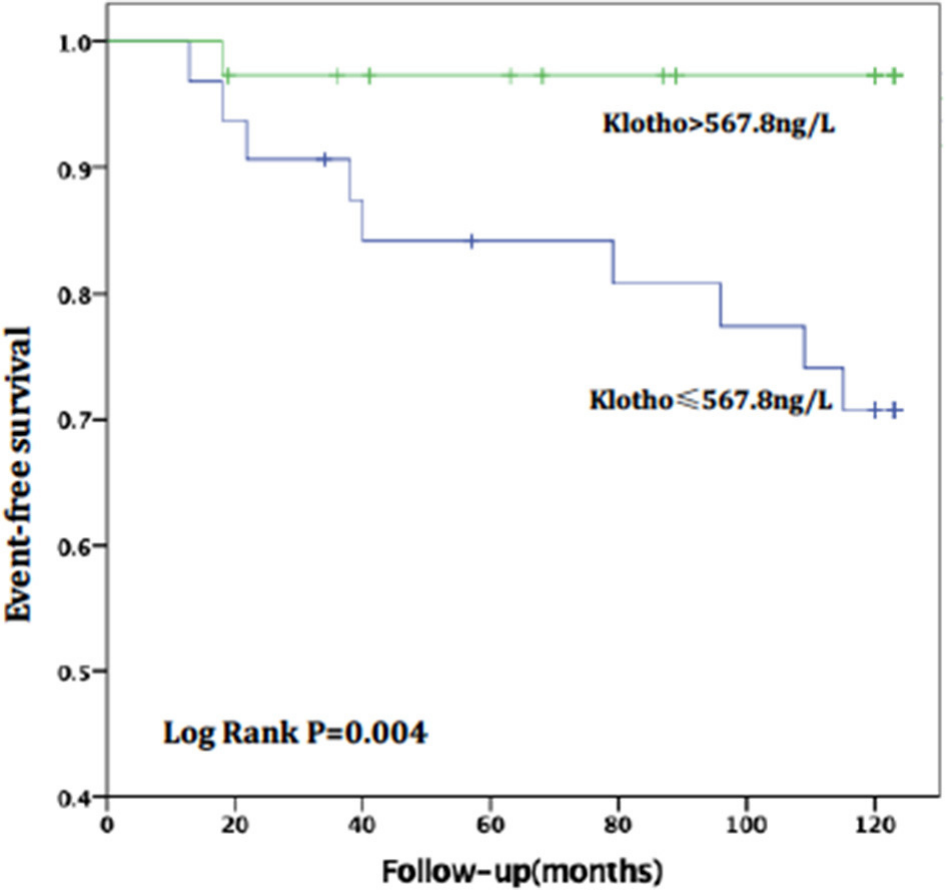
AAC ≤ 4, relationship between soluble Klotho level and CVD mortality in MHD patients. For patients with no or mild calcification (AAC ≤ 4), the high level of soluble Klotho level (sKl > 567.8 ng/L) patients had a lower risk of CVD death than in those with low level of soluble Klotho level (sKl ≤ 567.8 ng/L). Kaplan-Meier analysis with log-rank test revealed a significant difference between groups (*P* = 0.004).

**Figure 5:**
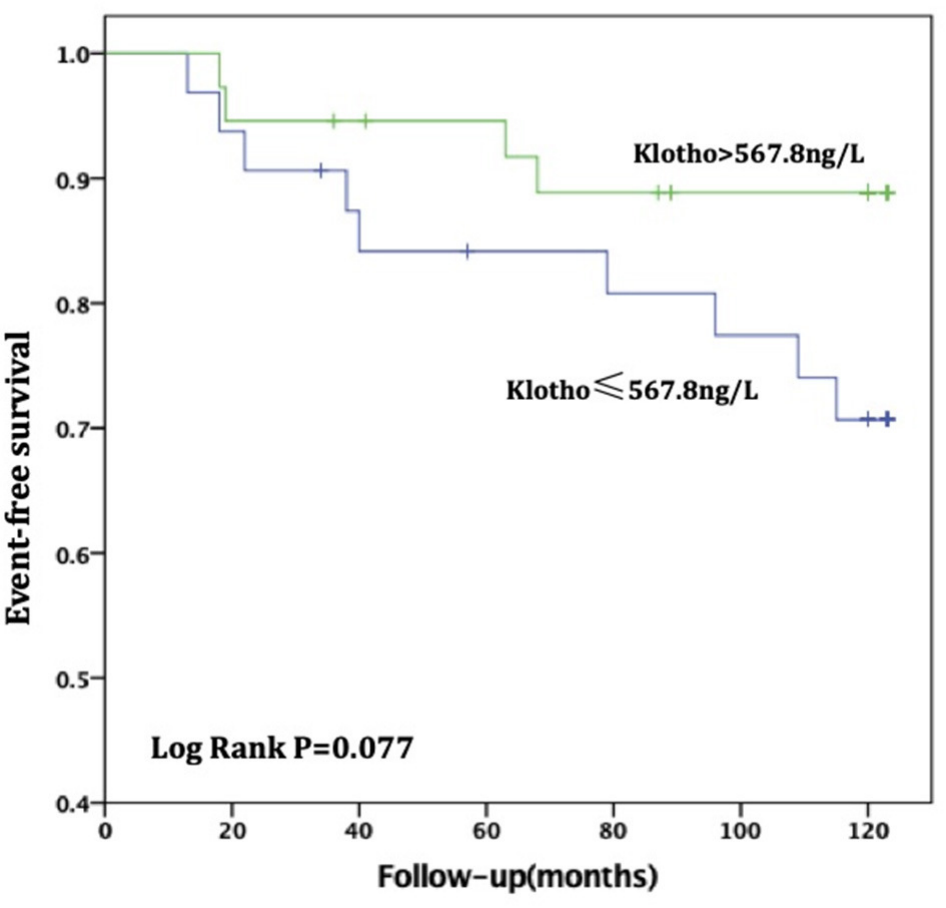
AAC ≤ 4, Relationship between soluble Klotho level and All-cause mortality in MHD patients. For patients with no or mild calcification (AAC ≤ 4), the high level of soluble Klotho level (sKl > 567.8 ng/L) patients had a lower risk of all-cause death than in those with low level of soluble Klotho level (sKl ≤ 567.8 ng/L). But Kaplan-Meier analysis with log-rank test revealed no significant difference between groups (*P* = 0.077).

For patients with no or mild calcification (AACs ≤ 4), the risk of all-cause death in the high sKL group was lower than in the low sKL group, but the difference had no statistical significance (*P* = 0.077). The risk of CVD death in patients with high sKL was significantly lower than in those with low sKL (*P* = 0.004). With mild or severe calcification (AACs ≥ 5), the risks of all-cause death and CVD death were not significantly different between the high and low sKL level groups (*P* = 0.706 and 0.488, respectively) (Figures 4, 5).

Cox regression analysis showed that for patients with no or mild calcification (AACs ≤ 4), lower levels of sKL [OR = 0.061 95% CI (0.008–0.483), *P* = 0.008] and age >60 years [OR = 9.863, 95% CI (2.062–47.168), *P* = 0.004] were independent risk factors for CVD death.

**Figure 6:**
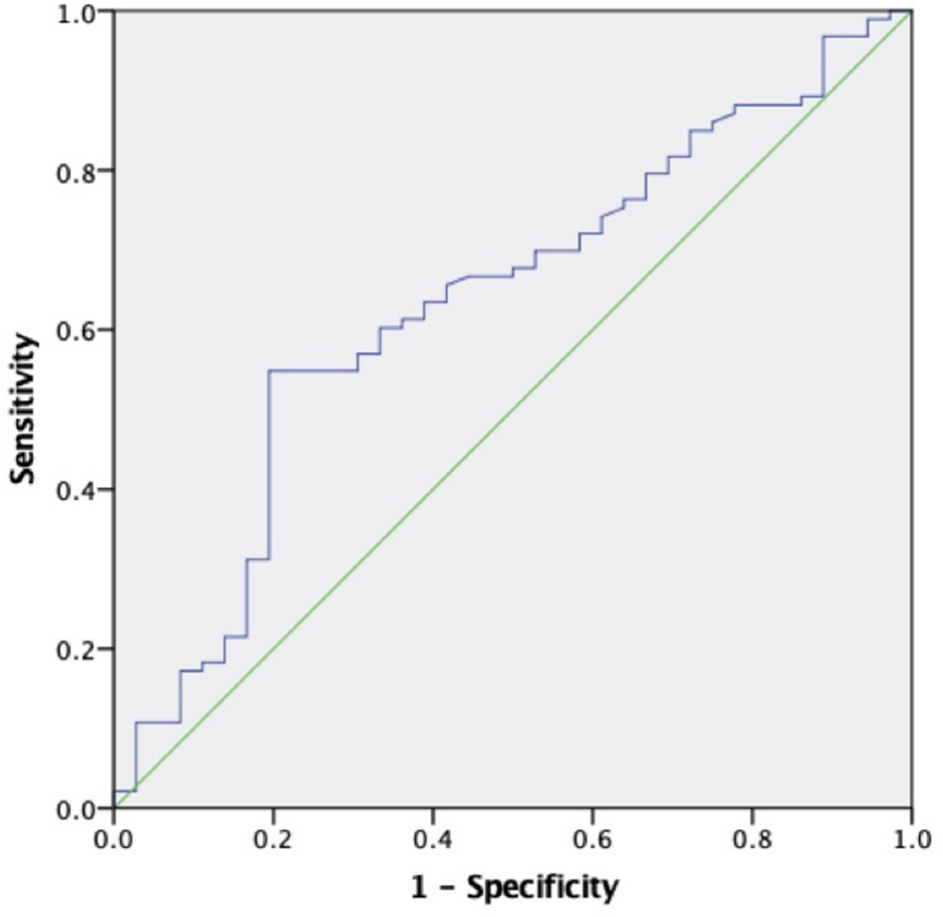
The role of soluble Klotho in predicting the CVD mortality in MHD patients. The receiver operating characteristic curve illustrates soluble Klotho. Areas under the curves are 0.634 (95% CI 0.528–0.740, *P* = 0.019) for the soluble Klotho. A cut off valuable of 566.52 pg/ml yielded to good sensitivity and specificity. The sensitivity and specificity are 57 and 69.4%, respectively.

**Figure 7:**
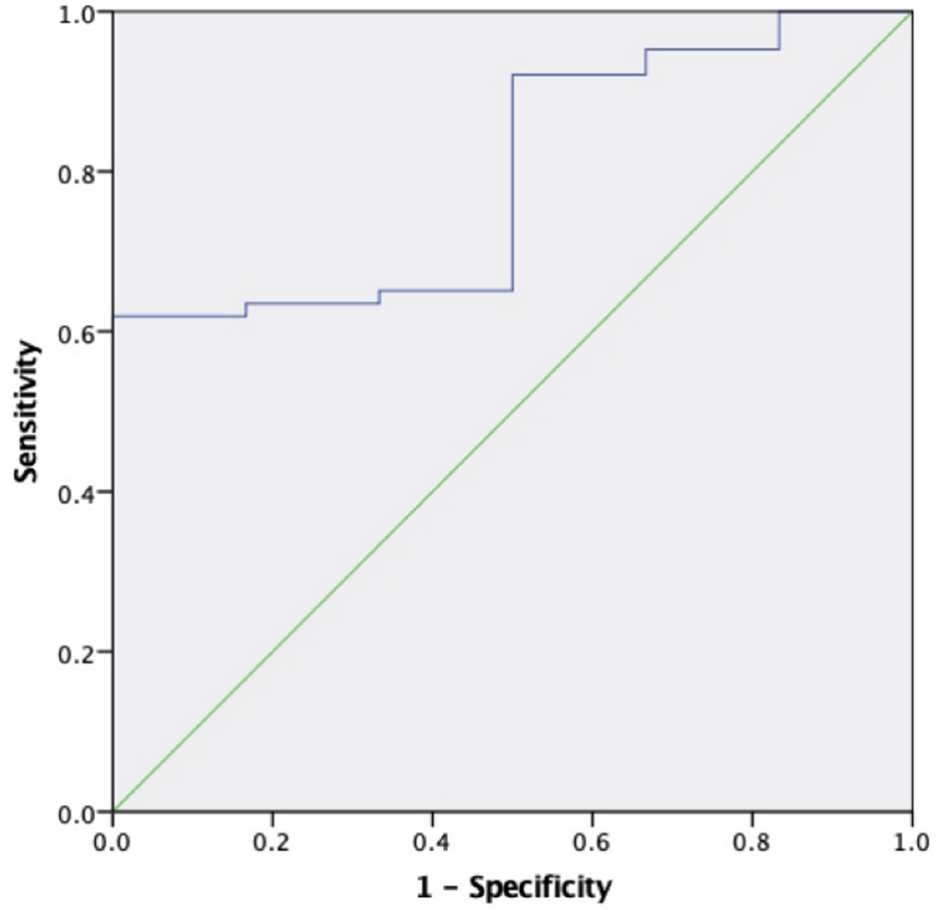
The role of soluble Klotho in predicting the CVD mortality in MHD patients with AAC ≤ 4. The receiver operating characteristic curve illustrates soluble Klotho. Areas under the curves are 0.796 (95% CI 0.647–0.946, *P* = 0.017) for the soluble Klotho. A cut off valuable of 307.69 pg/ml yielded to good sensitivity and specificity. The sensitivity and specificity are 92.1 and 50%, respectively.

The areas under the curve (AUC) for sKl to predict CVD death were 0.634 (95% CI 0.528–0.740, *P* = 0.019) and 0.796 (95% CI 0.647–0.946, *P* = 0.017) in MHD patients and in MHD patients with AACs ≤ 4 respectively, which showed that sKl concentration had a high accuracy for predicting CVD death especially in MHD patients with AACs ≤ 4. A cut off value of 566.52 and 307.69 pg/ml yielded to good sensitivity and specificity of predicting CVD death in MHD and MHD patients with AACs ≤ 4 by sKl level. The sensitivity and specificity were 57, 92.1, 69.4, and 50%, respectively. In MHD patients and in MHD patients with AACs ≤ 4, the AUC for sKl to predicting all-cause death were no significantly different between the high and low sKl level group (*P* = 0.484 and 0.397, respectively) (Figures 6, 7).

## Discussion

Mineral bone disease, which includes arterial calcification, is a common complication in CKD, especially in MHD patients. Vascular calcification is closely correlated with poor outcomes, including CVD death, in MHD patients. Klotho is an aging suppressor and encodes 130 kDa of Klotho protein. Klotho influences vascular calcification in CKD and directly inhibits vascular calcification. Klotho gene knockout animals exhibit a wide range of vascular calcification patterns and short life expectancy (3). sKL plays an important role in the prevention and treatment of acute kidney injury, reduces renal fibrosis induced by unilateral ureteral obstruction and slow the transition of AKI to CKD (9). There have been few studies on the relationship between sKL and prognosis in MHD patients. This study shows that sKL concentration can predict CVD death especially in MHD patients with no or mild abdominal aortic calcification.

Patients with low levels of sKL have a higher risk of CVD death, which may be related to vascular calcification in MHD patients. Animals lacking Klotho show upregulated expression of the phosphate transporters Pit1/2 and the key osteogenic transcription factor Runx2. Cells in high-phosphorus and uremic environments will upregulate their Pit1/2 activity. Both transporters can promote the influx of extracellular phosphorus into the cell, and promote cellular calcification (10). At the same time, Klotho can also release NO to reduce the vasoconstriction caused by FGF23 and phosphorus (11). Recently, studies have found that rapamycin can inhibit the mammalian target of rapamycin (mTOR) receptor to reduce vascular calcification and that it can also inhibit the mTOR-like receptor to increase the membrane and secretory Klotho concentrations. Importantly, rapamycin failed to reduce vascular calcification in the absence of Klotho by using either siRNA knockdown of Klotho or Klotho knockout mice, suggesting that Klotho may be in mediating the observed decrease in calcification by rapamycin *in vitro* and *in vivo* (12). In CKD patients, klotho knockdown potentiated the development of accelerated calcification through a Runx2 and myocardin-serum response factor-dependent pathway (13). The decreased level of soluble sKL in patients with CKD is an independent risk factor for vascular dysfunction, manifesting as arterial stiffness. Further studies are needed on whether CKD patients with arterial stiffness can be improved by Klotho supplementation (14). sKL level correlates with vascular calcification in MHD patients. Our previous studies found that sKL was closely related to abdominal aortic calcification in patients with MHD, and had diagnostic value in patients with severe calcification. Low levels of sKL were 4.5 times more prevalent than high levels in patients with moderate or severe abdominal aortic calcification (15). Therefore, Klotho is closely associated with vascular calcification and may be one of the reasons for vascular calcification in MHD patients.

Recently, studies have demonstrated that vascular calcification is closely related to CVD death. Among patients with coronary artery calcification, CVD death is 2.66 times more common than non-CVD death (16). Vascular calcification can be used as a predictor of CVD mortality in patients with MHD. Studies have shown that scores higher than 3 for vascular calcification in the pelvis indicate a 3.6-fold increase in CVD death risk, a 2.8-fold increase in cardiovascular hospitalizations and a 2.3-fold increase in non-fatal CVD events compared with scores below 3 (17). Calcification in other areas, such as the abdominal aorta and aortic arch, is an independent risk factor for CVD death in dialysis patients (18–20). The results of this study show that the risk of CVD death was significantly higher in the low sKL group than that in the high sKL group, especially the AACs was significantly low. Recently some studies have shown that high levels of soluble Klotho can reduce the risk of cardiovascular events and cardiovascular death by 61% in MHD patients compared with low levels of soluble Klotho patients. Even adjusted for age, gender, diabetes, cardiac function, dialysis vintage, serum hemoglobin, albumin, FGF23 and other factors, the risk still reduced by 14% (21, 22). However, these studies did not take into account the effect of vascular calcification on survival in MHD patients. Patients with low soluble Klotho may have severe vascular calcification (15, 23). Vascular calcification is a strong predictor of death in MHD patients (24). Therefore, these studies did not clarify the role of vascular calcification and soluble Klotho in predicting death in patients with MHD. Our study demonstrates that low soluble Klotho in Patients without or mild calcification can also predict cardiovascular death in MHD patients, which is a full complement and proof of previous studies.

Klotho may directly affect cardiac function. Hui et al. (25) showed that aging-related augmentation of inflammatory responses and cardiac dysfunction were associated with relative Klotho deficiency. Treatment with recombinant Klotho suppresses the inflammatory response and improves cardiac function in aging endotoxemia mice. In CKD, sKL can protect the myocardium from pathological stimuli, such as uremic toxins or FGF23 (26), even though myocardial hypertrophy still occurs in low sKL CKD mice in which phosphorus and FGF23 levels are controlled. Exogenous sKL can significantly improve cardiac hypertrophy in CKD mice. sKL may be a risk factor for cardiomyopathy in uremic patients, independent of FGF23 and phosphate (5). Klotho may inhibit apoptosis of cardiomyocytes by inhibiting the P38 and JNK signaling pathways (27). For patients with no or mild calcification in this study, the high risk of CVD death was significantly higher in the low sKL group compared with the high sKL group. It is suggested that sKL can affect the risk of CVD death independent of vascular calcification, and may directly affect and improve myocardial function.

This study has some limitations. First, vitamin D, which is regulated by FGF23 and Klotho, was not measured. The relationship between vitamin D, FGF23, and Klotho in the prognosis of MHD patients requires further study. Second, in the uremic environment, renal secretion of Klotho is reduced, and compensatory secretion is induced in other organs, such as the parathyroid glands, and cerebral choroid epithelial cells. The effect on patients' Klotho levels is unclear. However, animal studies have shown that parathyroid Klotho secretion decreased in uremia, suggesting that secretion may be reduced in all organs in uremia, but further studies are needed (28). Third, the sample size of this study is small, limiting the conclusions that can be drawn.

sKL and CVD death are closely related in MHD patients. A high sKL level is associated with low risk of CVD death, and may be an independent risk factor for CVD death in MHD patients. In MHD patients with no or mild calcification, low sKL levels have value in predicting CVD mortality. Soluble sKL concentration may be a biomarker that can predict CVD death in MHD patients especially with lower AACs.

## Data Availability Statement

The original contributions presented in the study are included in the article/supplementary material, further inquiries can be directed to the corresponding authors.

## Ethics Statement

The studies involving human participants were reviewed and approved by Ethics Committee on Human of Renji Hospital, Jiao Tong University. The patients/participants provided their written informed consent to participate in this study. Written informed consent was obtained from the individual(s) for the publication of any potentially identifiable images or data included in this article.

## Author Contributions

HC wrote the manuscript, conceived the study, and participated in its design. WZ and SM planned and supervised the study. JL, MZ, SL, and YZ collected and enrolled patients. ZN and LG reviewed and edited the manuscript. All authors read and approved the final manuscript.

## Conflict of Interest

The authors declare that the research was conducted in the absence of any commercial or financial relationships that could be construed as a potential conflict of interest.
